# Gel-Based and Gel-Free Identification of Proteins and Phosphopeptides during Egg-to-Larva Transition in Polychaete *Neanthes arenaceodentata*

**DOI:** 10.1371/journal.pone.0038814

**Published:** 2012-06-15

**Authors:** Kondethimmanahalli H. Chandramouli, Donald Reish, Pei-Yuan Qian

**Affiliations:** 1 Division of Life Science, Hong Kong University of Science and Technology, Hong Kong SAR, China; 2 Department of Biological Sciences, California State University, Long Beach, California, United States of America; Ecole Normale Supérieure de Lyon, France

## Abstract

The polychaete *Neanthes arenaceodentata*- is cosmopolitan in distribution-, has been used as a laboratory test animal. Life history of this species has several unique features; the female dies after spawning and the male incubates the fertilized eggs through the 21-segmented stage. The larvae leave the tube and commence feeding. Changes in protein abundance and phosphorylation were examined during early development of *N. arenaceodentata.* A gel-based approach and gel-free enrichment of phosphopeptides coupled with mass spectrometry were used to identify proteins and phosphopeptides in fertilized ova and larval stages. Patterns of proteins and phosphoproteins changed from fertilized ova to larval stages. Twelve proteins occurred in phosphorylated form and nine as stage specific proteins. Cytoskeletal proteins have exhibited differential phosphorylation from ova to larval stages; whereas, other proteins exhibited stage-specific phosphorylation patterns. Ten phosphopeptides were identified that showed phosphorylation sites on serine or threonine residues. Sixty percent of the identified proteins were related to structural reorganization and others with protein synthesis, stress response and attachment. The abundance and distribution of two cytoskeleton proteins were examined further by 2-DE Western blot analysis. This is the first report on changes in protein expression and phosphorylation sites at Thr/Ser in early development of *N. arenaceodentata.* The 2-DE proteome maps and identified phosphoproteins contributes toward understanding the state of fertilized ova and early larval stages and serves as a basis for further studies on proteomics changes under different developmental conditions in this and other polychaete species.

## Introduction

The polychaete *Neanthes arenaceodentata,* is cosmopolitan in distribution, and has been used as a toxicological test animal [1]. Life history of *N.arenaceodentata* has several unique features. Reproductive individuals of this species do not undergo mass spawning as swarming epitokes producing planktonic embryos such as many nereidids [2], but exhibit monogamous pairing. Female die after spawning. The male incubates, the fertilized eggs within a mucoid tube and is capable of reproducing multiple times [3]. Embryos remain within the male’s tube until the 21 segmented stage [4]. The embryos emerge from the fertilization membrane at about 10 days and development continues to the 21st segmental stage in 21–28 days. The larvae then leave the tube and commence feeding [4]–[5]. These unique changes must be controlled at the molecular level by the differential or specific expression of distinct sets of genes or proteins which coordinate and modulate various developmental events [6]. The molecular mechanisms that govern these changes are under translational/post-translational control [7]. Previous laboratory studies focused on toxicity [1] reproductive longevity [3], vitellogenesis in the developing oocytes [8], post-exposure feeding rate [9], and bioaccumulation of polychlorinated biphenyls [10] have been conducted on this species.Proteomic techniques have become useful tools in in recent years understanding developmental processes in polychaetes [11]–[13]. Proteomics applications cover many different aspects of development, including protein expression changes and phosphorylation dynamics during embryonic development [14]–[15]. Embryonic development in many marine polychaetes is a relatively rapid process and may be controlled by post-translational modification (PTM) of proteins [16]. Knowledge of protein modification is required to understand the cellular processes at the molecular level [17]. Multiplex proteomics technology permits quantitative, multicolor fluorescence detection of phosphoproteins and total proteins within a single gel electrophoresis experiment. Immobilized metal affinity chromatography (IMAC) coupled with LC-MS/MS provides an effective method for the determination of phosphorylation sites in a protein sample. However, despite rapid development in proteomics technologies and their application in polychaete larvae development [11]–[13], no such study has been conducted on protein expression and phosphorylation changes associated with embryonic development in *N. arenaceodentata*.

The purpose of this study was to examine protein expression changes during early development in *N. arenaceodentata* which has been raised in the laboratory for over 200 generations. In the present study, the proteome and phosphoproteome of fertilized ova, 3–4 segmented larvae and 10–12 segmented larvae of *N.arenaceodentata* were analyzed. A combination of multiplex 2-DE proteomics and mass spectrometry was used to identify differentially expressed or stage specific proteins by sequential fluorescence detection of proteins and phosphoproteins.

## Materials and Methods

### Specimen Culture and Sample Collection

**Figure 1:**
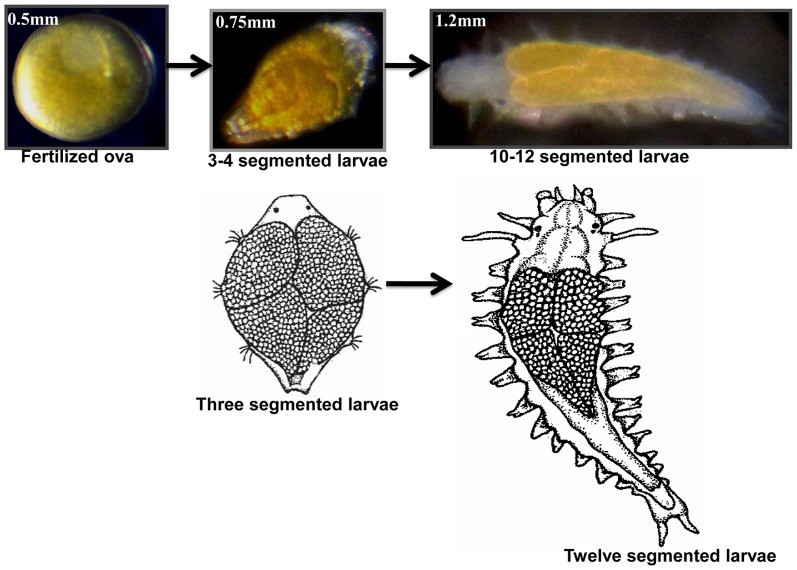
Early developmental stages of the polychaete *Neanthes arenaceodentata*. Three developmental stages were chosen for proteomic analysis: Fertilized ova, 3–4 segmented early larva and 10–12 segmented old larva. Drawings: Three segmented larva and twelve segmented larva.

The polychaete used in this study belongs to a species complex which is cosmopolitan in distribution. The California population is referred to as *N.arenaceodentata* and has been used as a toxicological test animal [1]. Specimens were taken from a laboratory population maintained at California State University, Long Beach. The culture was established in 1964 from 6 specimens collected from Los Angeles Harbor. No additional specimens have been introduced into the population which has undergone over 200 generations. Specimens from several matings were removed from tubes at fertilization, fertilized ova, 3–4 segmented early larvae and 10–12 segmented old larvae (Fig. 1) were shipped frozen to Hong Kong by overnight express.

Worms were washed through sieves with 0.2 µm filtered seawater (FSW) and examined under a microscope and debris removed. The samples were transferred to a lysis buffer (7 M urea, 2 M thiourea, 4% [3-[(3-cholamidopropyl)dimethylammonio]-1-propanesulfonate] (CHAPS), 40 mM dithiothreitol (DTT). Complete protease and phosphatase inhibitor was added to lysis buffer (Roche Applied Science, Mannheim, Germany) to prevent protein degradation and then frozen at −80°C.

### Preparation of Protein Samples and Two-dimensional Gel Electrophoresis

Sample preparation was carried out as described by Chandramouli *et al.* [13] with modifications. Eggs and larvae were sonicated on ice using10 sec blasts of 15% amplitude with 10 sec pauses between blasts. The samples were then centrifuged at 13,000 rpm for 10 min and the supernatant containing proteins were transferred to a clean centrifuge tube. Proteins were purified using a 2-DE cleanup kit (Bio-Rad, Hercules, CA). The purified protein pellets were resolubilized in lysis buffer and the protein concentration was determined using the modified Bradford method [18]_._ Two-hundred micrograms of each sample was sonicated for 10 min and incubated at room temperature for 1 hr for protein solubilization. Rehydration was carried out using 300µl of the sample in a buffer (7 M of urea, 2 M of thiourea, 4% CHAPS, 40 mM of DTT, 0.5% pI 4–7 ampholyte, and 1% bromophenol blue) on 17 cm immobilized pH gradient (IPG) strips (pH 4–7) for 14–16 hr. The samples were then subjected to isoelectrical focusing (IEF) using a Protean IEF Cell (Bio-Rad, Hercules, CA). Focusing was carried out at 250 V for 20 min and then along a gradient from 1,000 to 8,500 V to give a total of 60,000 Vh. Two-dimensional SDS-PAGE was conducted after the reduction and alkylation of the IPG strips with 2% DTT and 2.5% iodoacetamide (IAA).

### Multiplex Fluorescent Gel Staining and Image Analysis

The 2-DE gels were fixed 2–3 hr in 40% methanol and 10% acetic acid. The 2-DE gels were then incubated for 3 hr in ProQ Diamond (Invitrogen, Eugene, OR) to stain the phosphoproteins and destained with 20% acetonitrile (ACN) in 50 mM of sodium acetate (pH 4.0) for 3 hr. The destained gels were scanned for phosphoprotein spots using a Typhoon trio imager (GE Healthcare, Piscataway, NJ) at an excitation of 532 nm with a 610 BP 30 emission filter. The gels were incubated overnight in the dark with Sypro Ruby (Invitrogen, Eugene, OR) for total protein detection. They were scanned again using the Typhoon trio imager at an excitation of 582 nm with a 610 BP 30 emission filter. The gels were destained in 10% methanol and 7% acetic acid for 1 hr. The gels were stained with the modified G-250 Colloidal Coomassie Blue for protein spot excision and mass spectrometry analysis (MS). Three independent biological replicate gels were stained for phosphoproteins and total proteins and grouped and the protein patterns compared. Quantitative and qualitative analyses were carried out using the PDQuest software (Bio-Rad, Hercules, CA) as described by Thiyagarajan *et al.* [19]. Spots present in all three replicate gels were analyzed. A 1.5-fold threshold was set for quantitative detection of protein changes between three developmental stages. Protein and phosphoprotein spots which were significantly different (Student’s *t*-test, *p*<0.01) in successive stages were considered to be regulated.

### Phosphatase Treatment

Early segmented larvae were washed with FSW and sonicated in lysis buffer. Supernatant proteins were purified using a 2-DE cleanup kit. The purified protein pellets were resolubilized in lysis buffer and the protein concentration was determined by modified Bradford method [18]_._ The protein pellet was resuspended in lysis buffer to obtain a final concentration of 5 mg/mL. Lambda protein phosphatase treatment (λ-PPase) (New England Bio labs, Ipswich, MA) was performed on three biological replicates with modifications as described [20]. Two aliquots of 50 µL (250µg) protein sample were mixed with 5 µL of 10% SDS and vortex for 20 s. Each sample sequentially received 345 µL of deionized water, 50 µL of 20 mM MnCl_2_ and 50 µL of λ-PPase buffer. One aliquot was incubated with 400 units of λ-PPase and both samples were incubated with gentle agitation overnight at 30°C. Phosphatase-treated and untreated protein samples were purified by 2-DE cleanup kit and resuspended in rehydration buffer. IEF and 2-DE were performed as described in Chandramouli *et al.* [13]. The 2-DE gels were sequentially stained for phosphoproteins and total proteins with ProQ Diamond and Sypro Ruby. The gels were scanned using a Typhoon trio imager.

### Protein Identification from 2-DE

Twenty-six stage specific and differentially expressed protein and phosphoprotein spots were analyzed by MS. Each protein was inspected for specific and differential expressions to match the PDQuest software spot detection and to ensure the selected spots were reproducibly detected among three replicates. The protein spots were excised, washed, and digested in 20 µL of 12.5 ng/mL trypsin (Promega, Madison, WI) in 10% acetonitrile and 10 mM of NH_4_HCO_3_ at 37°C for 16 hr. The peptides were extracted and dried in a speed vacuum as described by Chandramouli *et al*. [13].

Each of the dried peptide samples was reconstituted in 10 *µ*L of 0.1% formic acid. The peptides were cleaned using C18 Zip Tip columns (Millipore, Billerica, MA) and analyzed using a nanoflow UPLC (nanoAcquity, Waters) coupled with an ESI-hybrid Q-TOF (Premier, Waters) tandem mass spectrometer. The Q-TOF was set to perform data-dependent acquisition in the positive ion mode with a selected MS survey mass range of 300–1600 *m*/*z*. The three most abundant peptides with +2 to +4 charge above a 40-count threshold were selected for MS/MS as described in Zhang *et al* [11]. The raw MS data were searched against a NCBI nr and Swiss-Prot database. MS/MS spectra were also matched against in-house transcriptome databases of the polychaete; *Hydroides elegans, Pseudopolydora vexillosa* and protein databases of *Capitella* sp.I to improve the accuracy of protein identification rate. The transcriptome databases were constructed by 454 sequencing of transcriptome of *H.elegans and P.vexillosa*. The *P.vexillosa* database contained 55,831 unique sequences, of these 4,358 were contigs and 89,532 remained as singletons (12). The MS raw data were converted into peak list files in *pkl* format using proteinlynx (version 2.2.5, Waters) (smooth 3/2 SavitzkyGolay and center 4 channels/80% centroid). Mascot was set up to search respective databases. One missed cleavage was allowed for trypsin digestion. The mass tolerances were set at 30 ppm for the peptide precursors and 0.5 Da for fragment ions. Carboxamidomethylation at cysteine residues were set as fixed modification, and oxidation at methionine, phosphorylation at serine, threonine, or tyrosine were set as variable modifications. All identified proteins had protein scores >51 for NCBI nr, and >39 for Swiss-Prot databases and individual ion scores were >20. A MOWSE threshold for proteins *P* value of <0.05 was considered to be statistically significant**.** Mascot protein score values are listed in Table 1.

**Table 1:** Identification of abundant proteins during early development in *Neanthes arenaceoentata.*

Spot No.	Acc No.a)	Protein nameb)	Score	MWc)	pId)	PM/SC	Peptide sequence
1	gi|116057804	Myosin class II heavy chain	82	97/46	6.1/6.0	18/4	*K VMSLVAR QKADDEL DR* *LTG**S**EDTIR
2	gi|116057804	Myosin class II heavy chain	104	97/46	6.1/6.0	13/8	*SKSSNIFKGLLLGVDMRK DA ALDEQRK* *L**T**GSEDTIR
3	gi|124394878	Unnamed protein product	89	97/57	6.0/8.9	6/15	*NNAIITTK EEQLYQKK MMQ MEEEK*
4	gi|291223597	Conserved hypothetical protein	102	40/15	6.0/6.6	11/13	*IPFEEFQK KFEELIK*
5	Q48UF3_STRPM	Putative extracellular matrix binding protein	105	40/229	5.0/6.5	17/8	*GTWEVSADGSSGR DSYV KITK*
6	RL29_BOVIN	60 S ribosomal protein L29	92	40/17	5.2/9.0	6/36	*NGI KKPR YES LKGVDPK*
7	gi|86559737	Alpha-tubulin	199	44/48	4.6/5.8	6/23	*EDAANNYAR EHIDIVLDR*
8	gi|312861909	Non-muscle actin II	210	28/46	6.0/5.2	6/19	*AG FAGDDAPR DSYVGDEAQS KR*
9	Q2U497_ASPOR	Myosin class II heavy chain	Hypothetical protein	29/277	4.9/6.0	13/18	*K VMSLVAR QKADDEL DR*
10	gi|71663765	Hypothetical protein	Actin-15A	18/20	5.7/5.4	15/14	*HSGGGD THYKMCK AFR EEEEK* *NGSLSK**T**R
11	gi|33642225	Actin-15A	159	20/42	5.3/5.2	5/14	*A GFAGDDAPR DSYVGDEAQ SKR*
12	Q48UF3_STRPM	Putative extracellular matrix binding protein	89	229/18	6.5/4.8	7/12	*MYAFNSGPRS CR NPGWH EHNK*
13	Q16UG5_AEDAE	Centrosomal protein	100	227/18	5.9/4.8	8/15	*QNNELR QRLQEEASTYR DHSPGR*
14	gi|66547450	60 kDa heat shock protein	104	60/66	5.6/5.3	2/13	*E LVESSAKLSK KSGDGSEAK*
15	gi|56069835	Beta-tubulin	457	42/46	6.1/4.7	5/13	*INVY YNEATGGK AVLVDLEP GTMDSVR* *AVLVDLEPGTMD**S**VR
16	gi|20069089	Alpha-tubulin-2	199	60/50	4.7/4.7	9/26	*EIVDLVLDR LIGQIVSSITASLR*
17	gi|33642225	Actin	96	41/42	5.5/5.3	2/5	*GYS FTTTAER EITA LAPSTMK*
18	gi|312861909	Beta-actin	246	41/42	5.5/5.2	7/24	*AGFAGDDAPRAVFPSIVGR VAPEE HPVLLTEAPL NPK*
19	gi|20069089	Alpha tubulin	199	50/55	4.9/5.2	5/17	*EIVDLVLDR LIGQIVSSITASLR*
20	Q2LDZ6_HIRME	Cytoplasmic actin	246	42/44	5.1/5.4	5/29	*AG FAGDDAPR DSYVGDEAQS KR*
21	gi|33642225	Beta-actin	428	42/42	5.3/5.3	4/15	*AG FAGDDAPR DSYVGDEAQS KR*

a) Accession numbers are from NCBI nr and Swiss-Prot database.

b) significance threshold level for positive identification was p<0.05.

c) observed MW and p*I* values were estimated from 2-DE gels.

d) Theoretical MW and p*I* values were derived from a database search by MASCOT. PM: number of peptides matched to protein sequence; SC: sequence coverage.

* phosphopeptides and phosphorylation sites are underlined.

### Phosphopeptides Identification by LC-MS/MS Analysis

Early 3–4 segmented larvae were washed with FSW and sonicated in lysis buffer (7 M urea, 2 M thiourea, 4% CHAPS). Supernatant proteins were purified and concentration was determined as described [18]_._ One milligram of protein was resuspended in a buffer containing 8 M urea, 50 mM ammonium bicarbonate pH 8.0, reduced with 5 mM DTT for 30 mins at 37°C and alkylated by 20 mM iodoacetamide. It was incubated in the dark for 1 hr at room temperature then diluted 7-fold with 50 mM of NH_4_HCO_3_. In-solution digestion was done with trypsin at an enzyme/protein ratio of 1/50 and incubated for 16 hours at 37°C. The resulting digest was desalted using Sep-Pak C18 cartridges (Waters, Milford, MA), and dried in a Speed Vac (Thermo Electron, Waltham, MA).

### Affinity Enrichment of Phosphopeptides

The peptides were reconstituted in 250 mM acetic acid with 30% acetonitrile (wash/equilibration solution) and pH adjusted to 2.5–3.0. PHOS-Select Iron Affinity Gel beads (Sigma, St. Louis, MO) were equilibrated with equilibration solution before sample loading. The peptide samples were loaded into the IMAC column and incubated and mixed for 30 min. Unbound peptides were removed with 500 µl wash solution, followed by an affinity gel wash with deionized water. The bound peptides were eluted with 200 µl of elution solution (400 mM ammonium hydroxide). Eluted peptide samples were dried under vacuum and then reconstituted in 0.1% (v/v) FA for further desalting and concentration using C18 Zip Tips (Millipore, Bedford, CA).

### LC-MS Analysis

The samples were concentrated in a peptide trap (Waters) and then analyzed using a nanoflow UPLC (nanoAcquity,Waters) coupled with an ESI-hybrid Q-TOF (Premier, Waters) tandem mass spectrometer as described in Zhang *et al.* [11]. Mascot was set up to search the NCBI nr database assuming trypsin as the digestion enzyme. The mass tolerances were set at 20 ppm for the peptide precursors and 0.5 Da for the fragment ions. The false discovery rate (FDR) of protein identification was evaluated by repeating the search using identical parameters against a randomized decoy database created by Mascot. FDR was controlled to <2%.

### SDS-PAGE and 2-DE Western Blot Analysis

The 2-DE Western blot was performed to confirm the abundance and distribution of the tubulin and actin isoforms on gels following described protocol [12]. Equal amounts of protein lysates (75 µg) from ova and larval stages were subjected to IEF using 7 cm IPG strips with a linear pH 4–7 gradient (Bio-Rad, Hercules, CA). They were separated electrophoretically on 12.5% (8×7.3 cm2) SDS-PAGE and transferred to an immobilon PVDF membrane (Millipore Corporation, Bedford, MA). The membranes were blocked, and incubated for 16 hr at 4°C with antibodies of anti-alpha/beta-tubulin rabbit, (Cell Signaling, Danvers, MA) and anti-actin clone C4 mouse monoclonal (Millipore, Billerica, MA) at a dilution of 1∶2000. The membranes were incubated with corresponding HRP conjugated secondary antibodies at a dilution of 1∶5000 for 1 hr and developed using an ECL western blotting analysis system (Millipore, Billerica, MA).

Total protein expression levels of three selected proteins (tubulin, actin and HSP90) were performed by Western-blot analysis following the protocol described by Chandramouli *et al*. [13]. Ten µg of protein lysates from ova and larval stages were separated on 12.5% SDS-PAGE and transferred onto immobilon PVDF membrane. The membranes were incubated with 1∶2000 diluted monoclonal antibodies of anti-alpha/beta-tubulin rabbit, anti-actin, clone C4, mouse monoclonal and anti-hsp-90 rabbit (Cell Signaling, Danvers, MA) for ∼16 hr at 4°C. The membranes were probed with horseradish peroxidase-conjugated secondary antibodies (1∶5000 dilutions) for 1 hr at room temperature and developed as decribed by Chandramouli *et al*. [13].

## Results

### Two-dimensional Proteome Maps of Early Developmental Stages of *Neanthes Arenaceodentata*

**Figure 2:**
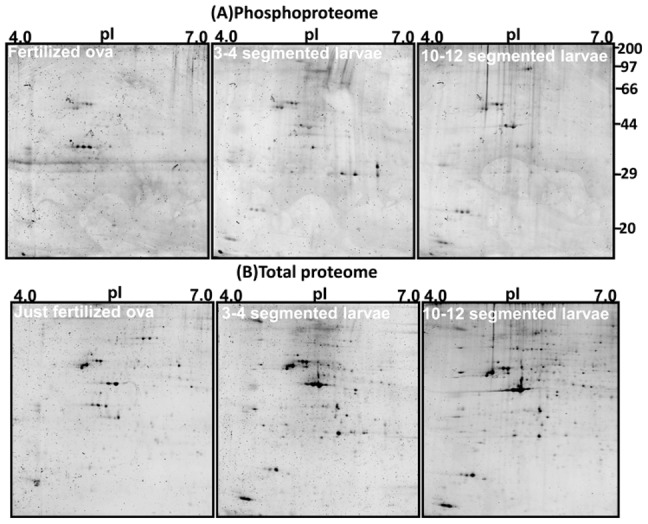
Representative proteome and phosphoproteome 2-DE gel images of fertilized ova, and two larval stages of *Neanthes arenaceodentata*.

**Figure 3:**
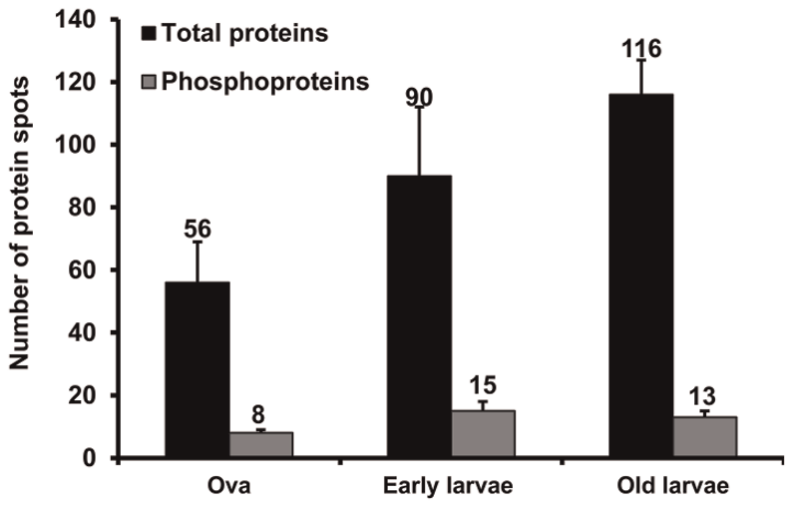
The number of protein and phosphoproteinspots reproducibly detected in fertilized ova, and larvae of *Neanthes arenaceodentata.*

**Figure 4:**
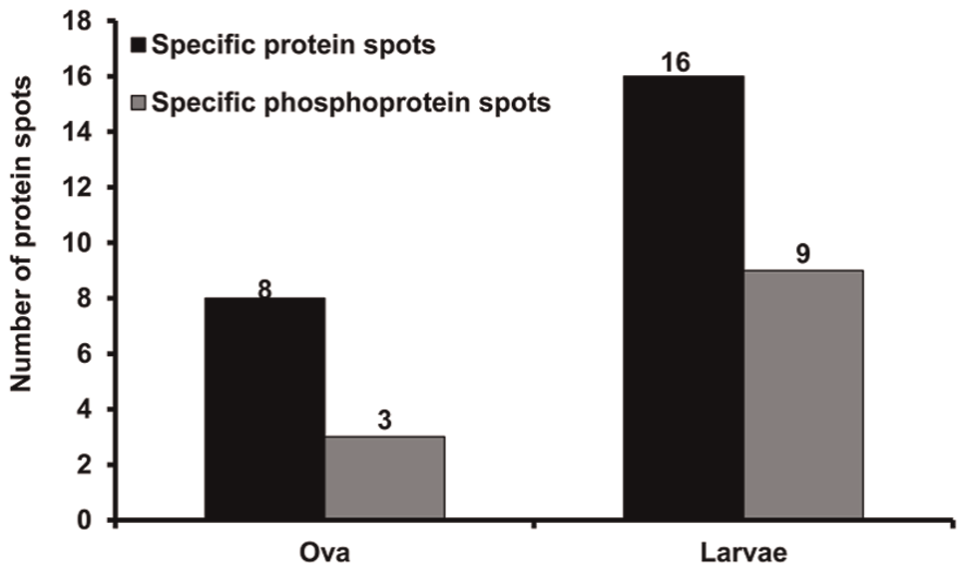
The number of differentially expressed abundant total protein spots and phosphoprotein spots in fertilized ova, and larvae. Differentially expressed spots showed significant differences between three stages (Student’s *t*-test *p*<0.01, n = 3).

**Figure 5:**
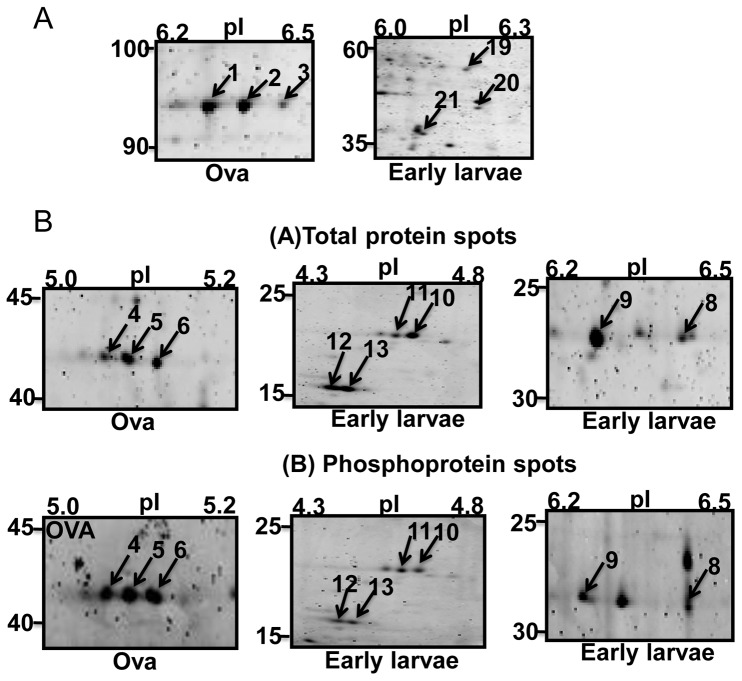
A close view of stage specific total proteins (A) and phosphoproteins (B) expressed in fertilized ova, and larvae of *Neanthes arenaceodentata.*

**Figure 6:**
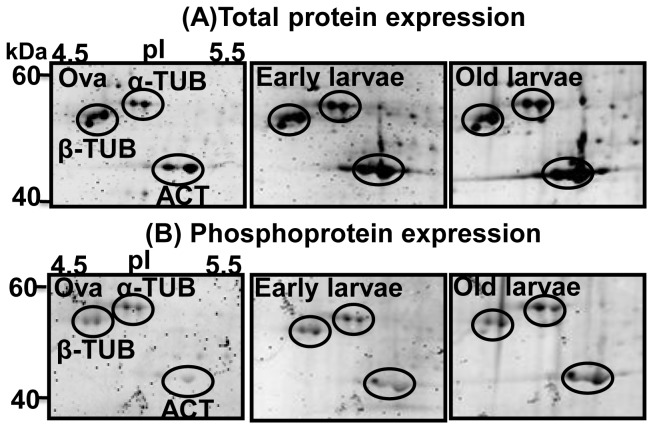
A close view of differential phosphorylation of cytoskeleton proteins (spots marked in circle) in fertilized ova and larvae of *Neanthes arenaceodentata.*

The proteome and phosphoproteome gels of the early developmental stages (Fig. 1) were analyzed by PDQuest software. Reproducibility of 2-DE is affected by gel to gel variation among replicate gels and variability in the biological material used. Sample preparation and subsequent 2-DE was performed in three independent biological replicates and one technical for good reproducibility among replicate gels. Fig. S1 and Fig. 2 show consistent pattern of abundant protein spots of proteome and phosphoproteome between replicate gels. The ProQ diamond stained phosphoproteome gels of fertilized ova (OVA), 3–4 segmented early larvae and 10–12 segmented older larvae are shown in Fig. 2; upper panel. The PDQuest analysis of three independent replicate gels detected 8, 15 and 13 phosphoprotein spots from the ova, early larvae and older larvae respectively (Fig. 3). After phosphoprotein staining, the 2-DE gels were stained with SYPRO Ruby. They were used as reference gels to compare protein patterns in the same gels (Fig. 2; lower panel). The gel analysis indicated that 56, 90, and 116 total protein spots were detected from the ova, and the two larval stages, respectively (Fig. 3). The pattern of protein and phosphoprotein changed from the ova to larval stages; whereas, the protein expression profile of the two larval stages appeared similar with minor differences. The protein and phosphoprotein spots increased from the ova to the two larval stages. Eight stage-specific proteins were detected in the ova; of which three were phosphoproteins. Sixteen abundant stage-specific total proteins were detected in larval stages, of which 9 were phosphoproteins (Fig. 4). Enlarged 2-D gels of specific proteins and phosphoproteins are shown in Fig. 5A and 5B. Many protein spots commonly expressed and differentially phosphorylated were analyzed in all three stages (Fig. 6 spots marked in circle).

### Identification of Commonly Expressed and Stage Specific Proteins by ESI-QTOF

Proteome analysis in non-model species with limited or lack of genome information is difficult since as sequence information is required to identify proteins by matching peptides in MS spectra against known protein sequence databases. A total of 21 proteins, 12 of which occurred in phosphorylated form and 9 as stage specific or differentially expressed spots were identified (Table 1). The observed molecular mass (MW) and isoelectric points (pI) of the identified proteins were close to the theoretical values derived from the MASCOT database. However, the MWs and pI values of proteins spots 1–6, 8, 9 and 11–13 in the gel deviated from these values suggesting that they were post-translationally modified during early development. Many of the abundant proteins identified in the NCBI nr and Swiss-Prot database were also identified from the transcriptomic database with high confidence scores (Table S1), reflecting the accuracy of the protein identification. Several protein spots identified appeared as abundant cytoskeleton proteins in the 2-DE gels, including actin, tubulin and myosin. Some cytoskeleton proteins appeared as the same protein or isoforms, such as alpha-tubulins (α-TUB) (spots 7, 16,and 19), beta-tubulins (β-TUB) (spot 15), actins (ACT) (spot 17), beta-actins (β-ACT) (spots 8, 18, 21), actin, cytoplasmic A3 (ACT-CA3) (spot 20), actin-15A (ACT15) (spot 11), myosin class II heavy chain (ISS) (spots 1,2 and 9) and centrosomal protein (CP) (spot 13). Sixty percent of the identified proteins were related to structural reorganization during early stages of worm development. Identified proteins were also related to protein synthesis, including 60 S ribosomal protein L29 (RP) (spot 6), extracellular matrix binding protein (EMBP)(spots 5 and 12) and heat shock protein 60 (HSP60)(spot 14). Three abundant hypothetical proteins (HP) were identified whose expression was stage specific (spots 3, 4: Ova and spot 10: larvae). The function of these “conserved” hypothetical proteins has not been characterized although they may play a role during the early development.

### Differential and Specific Phosphorylation of Proteins During Early Development

Cytoskeletal phosphoproteins such as β-TUB (spot15), α-TUB (spot16), ACT (spot17) and β-ACT (spot18) showed a gradual increase in phosphorylation from ova to larval stages (Fig. 6 spots marked in circle). To determine if the variations in phosphorylation levels in the three stages were due to changes in the total protein expression, comparisons were made of the total protein expression on the same gels. The changes in the phosphorylation level of actin and tubulins were due to their protein expression changes. Three proteins or protein isoforms, HP (spot 4), EMBP (spot 5) and RP (spot 6) exhibited stage-specific phosphorylation patterns in the ova stage. Whereas in the two larval stages, ISS (spot 9), ACT (spot 8), EMBP (spot 12) and CP (spot13) indicated stage-specific phosphorylation patterns (Fig. 5B).

### Dephosphorylation of Proteins by λ-PPase

**Figure 7:**
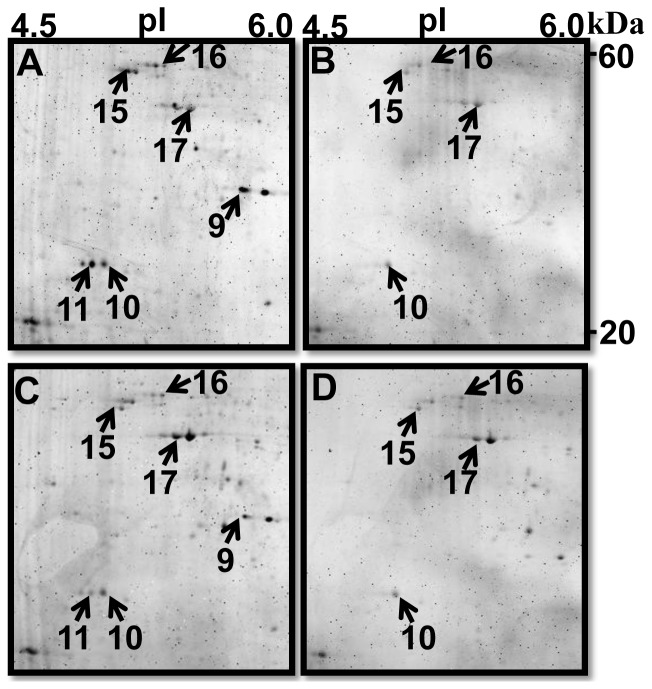
Close-up images of phosphorylated and dephosphorylated spots. 300 µg Proteins of 3–4 segmented early larvae were incubated without (A) or with (B) 400 U λ-PPase and separated by 2-DE (pH range 4–7). The upper and lower rows show the spot pattern in the absence (A and C) or presence (B and D) of λ-phosphatase. Phosphorylated proteins (arrow heads) were detected by Pro-Q Diamond phosphoprotein stain (A and B) and SYPRO Ruby total protein stain (C and D). Spot numbers are corresponding to proteins listed in Table 1.

The enzymatic treatment resulted in the loss of phosphate groups in protein spots (Fig. 7). The λ-PPase activity was efficient and only faint protein spots were detected in the sample treated with λ-PPase (Fig. 7B and 7D). Spots 10, 15–17 showed decreased phosphorylation level of protein spot upon treatment which corresponded to an increase in phosphorylation of the non-phosphorylated protein spots (Fig. 7A and 7C). Spots 9 and 11 nearly disappeared in both ProQ Diamond (Fig. 7B) and Sypro Ruby (Fig. 7D) stained gels. Among six protein spots, spots 10 and 16 displayed a basic p*I* shift of approximately 0.1 pH units after λ-PPase treatment. These six spots indicated by the arrows in Fig. 7 were excised, in-gel digested and subjected to ESI-Q-TOF mass spectrometer. Proteins were identified as myosin class II heavy chain (spot 9), hypothetical protein (spot 10), actin (spot 11 and 17) and tubulin (spot 15 and 16) as listed in Table 1.

### Phosphopeptides Identification by LC-MS/MS

Because of the limitation of a gel-based proteomic approach to identify low abundant phosphoproteins, the shot-gun proteomics approach was employed to enrich larval phosphopeptides on IMAC followed by LC-MS/MS analysis. 10 phosphopeptides were indentified (Table 2). Equal proportions of phosphorylation on serine or threonine were detected on the identified phosphopeptides. These phosphopeptides belonged to nine proteins, including ISS, ACT, ATP synthase beta subunit (ATPase), pyruvate dehydrogenase (PD), CBR-RIC-8 protein (CBR8), signaling inositol polyphosphate 5 phosphatase(SIP), GK20533 (GK) and mothers against decapentaplegic-like protein 6 (MAD6). Three phosphoproteins were identified from both gel-based and gel-free approach (Table 1 and 2), suggesting that these are probably the most abundant phosphoproteins in larvae. Cytoskeleton proteins ISS and ACT were found to be phosphorylated at Thr-628, Thr-107 and Thr-298 respectively. The PD was found to be phosphorylated at Ser-289 and Ser-291, ATPase at Ser-242, CBR8 at Thr-69, SIP at Thr-1173, GK at Ser-16 and Ser-50 and MAD6 at Thr-214 and Ser-230. The mass spectra of the phosphorylated peptides^625^YSGHSMSDPGTSYR^640^ for PD and ^285^MLFTPPEDTSPAGGKK^298^ for ISS are shown in Fig. S2.

**Table 2:** Identified phosphorylated proteins and phosphorylation sites in 3–4 segmented larvae by IMAC enrichment followed LC-MS/MS.

Accession number	Protein	Fragment	Phosphopeptides	Designation
gi|315439552	myosin heavy chain	625–640	MLF**T**PPEDTSPAGGKK	Thr-628
gi|71370860	ATP synthase beta subunit	234–247	FTQAGSEV**S**ALLGR	Ser-242
gi|313507212	beta-Actin	100–114	EHPVLL**T**EAPLNPK	Thr-107
gi|3182894	actin	292–313	KDLYAN**T**VLSGGTTMVPGIADR	Thr-298
gi|129048	pyruvate dehydrogen ase,mitochondrial	285–298	YSGHSM**S**DPGTSYR YSGH**S**MSDPGTSYR	Ser-291 Ser-289
gi|268553585	C. briggsae CBR-RIC-8 protein	58–72	IDNDDLASILLE**T**VR	Thr-69
gi|1277082	signaling inositol polyphosphate 5 phosphatase	1166–1180	SEINQQ**T**PPTPTP	Thr-1173
gi|195441509	GK20533	16–51	**S**GSTGSANINLMSGVAGGGGG-GGGGAAAAASASA**S**K	Ser-16 Ser-50
gi|332021148	mothers against decapentaplegic-like protein 6	211–233	SQG**T**EAVGACRLPLTLAY**S**PR	Thr-214 Ser-230

The phosphorylation sites are underlined.

### Distribution of Cytoskeleton Protein Isoforms and its Abundance in Early Developmental Stages

**Figure 8:**
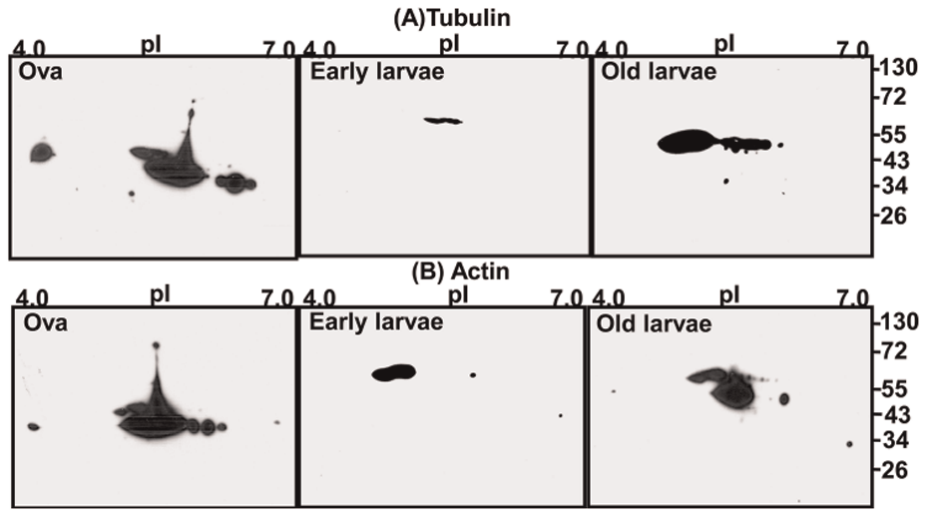
Two-dimensional Western blot analysis of tubulin (A) and actin (B) in Ova, early larvae and old larval stages of *Neanthes arenaceodentata*.

**Figure 9:**
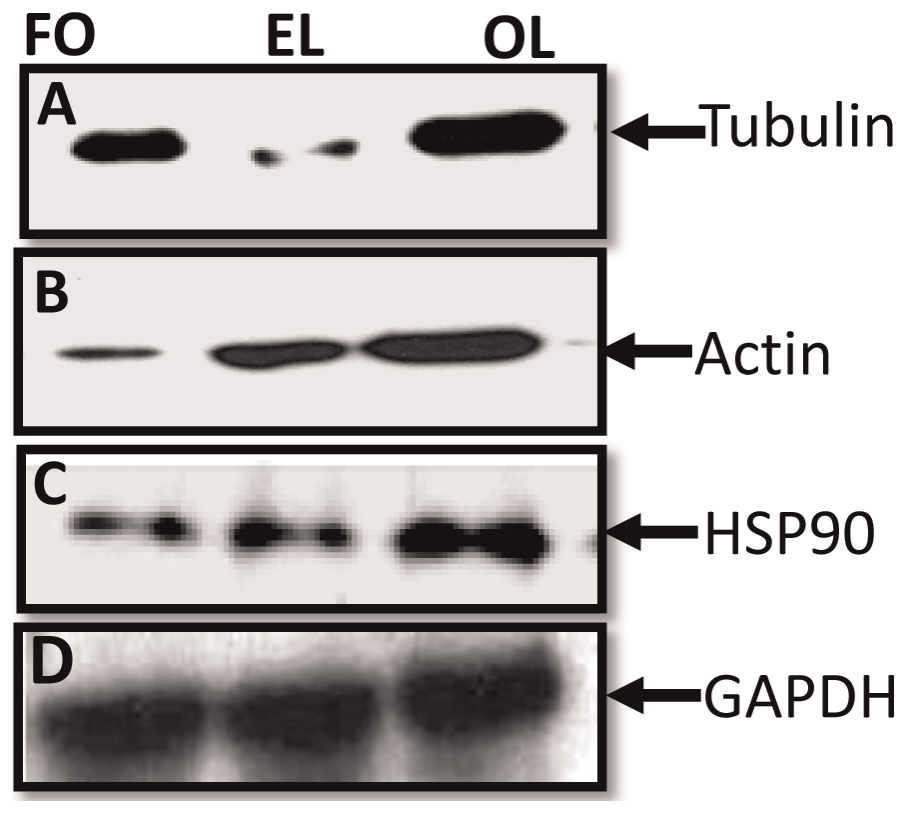
Western blot analysis of tubulin (A), actin (B) and HSP-90 (C) in Ova, early larvae (EL) and old larval (OL) stages of *Neanthes arenaceodentata*.

Antibodies against proteins are not commercially available for this species. Therefore, three conserved proteins were selected for Western blot analysis. Sixty percent of the identified proteins spots were isoforms of cytoskeleton proteins such as tubulin and actin and had different MW and pI values. The 2-DE Western blots indicated that many of the isoforms of tubulin (Fig. 8; upper panel) and actin (Fig. 8; lower panel) had MW and pI values (tubulin: Mr ∼72-34/pI 4.5-6.2 and actin: Mr ∼72-34/pI 4.0-7.0)**.** Both the α-TUB (Mr ∼55-43/pI 5.5-6.0) and β-TUB (Mr ∼55-43/pI5.5-6.5) isoforms were present in all three stages. Tubulin isoforms abundance decreased in early larval stage, whereas the abundance was maintained in the late larval stage (Fig. 9A). A high degree of heterogeneity of actin isoforms was found in the ova and late larval stages indicating the presence of many different isoforms of actins such as β-ACT, ACT and cytoplasmic ACT 3. The isoforms expression appeared to decrease in the early larval stage. Isoforms expression was maintained in the older larval stage (Fig. 9). Interestingly, the total actin expression level increased from ova to larval stages (Fig. 9B). Cytoskeleton protein, HSP was also identified (Table 1, spot 14). The Western blot analysis using anti-Hsp90 antibody was used to verify the differential expression from 2-DE. The expression of Hsp90 increased from ova to larval stages as shown in Fig. 9C.

## Discussion

### Cross-species Protein Identification

Proteomic approaches are useful for studying early developmental stages where regulatory mechanisms are driven by protein expression changes and PTMs [21]–[22]. Several proteomic studies on polychaete have been recently published [11]–[12]. Although mitochondrial genome sequences have been reported for some marine invertebrates. [12], [14], [23]–[26], protein identification in non-model species is hindered by insufficient or lack of genome sequences. This is the first report providing molecular information at proteome level in *N.arenaceodentata*. The proteome of fertilized ova and larvae were chosen in order to document proteins which might regulate or mediate early development. A total of 21 different proteins and/or isoforms were identified by either conventional database searching of MS spectra against known databases or a combined approach which included MS spectra search against customized transcriptome databases of other polychaetes and MS-BLAST sequence similarity searches. Polychaete genome sequence information is incomplete; the failure to identify some protein spots was related to the lack of homologous protein sequences in the databases. A promising approach to overcome these problems is use of high-throughput transcriptome sequencing of non-model species [25], [26].

### Changes in Protein Expression Patterns and Phosphorylation of Proteins During Early Development

Ovulated egg is a unique transcriptionally quiescent cell [27] prepackaged with maternal mRNAs and proteins to facilitate the transition from egg to larval stages. This species of polychaete undergo early development from fertilization through the 21-segmented stage in the male’s tube. Dramatic changes in cellular physiology occur during the fertilized ova to early larval transition. The increase in protein and phosphoprotein spots from the ova to larval stages indicates rapid and significant changes in protein abundance and protein phosphorylation. In addition, specific expression of ova and larval stage proteins or isoforms, may indicate that specific protein functions are restricted to particular stages of early development. Very little transcription or translation is detectable [28], [29], translation increased 5-fold [29], but transcription is not required for development until late blastula stage [28]. Therefore, we believe that the specific ova proteins, such as 60 S ribosomal protein L29, hypothetical or novel proteins make up the protein synthesis machinery when new transcription and translation are needed in the early larval stages. Several hypothetical proteins were identified, whose expression was either specific to ova or larval stages. Although the function of these proteins has not been characterized, they probably play important role in egg activation and subsequent developmental processes.

### Cytoskeleton Proteins Abundance in Early Developmental Stages

The molecular events occurring during early development include changes in intracellular physiology and remodeling of the cytoskeleton [30], [31]. The cytoskeleton proteins are the basis of the organization of eukaryotic cells. In this study, 60% percent of the protein spots were identified as the structural proteins and appeared as abundant proteins in the 2-DE gels. Among them, tubulin is a main component of the cytoskeletal microtubules [32]. The role of spindle microtubules in early mitotic cell divisions during development is well documented; therefore, it was not surprising to find that α-TUB and β-TUB were abundantly expressed in the fertilized ova and larval proteome. These cytoskeleton proteins controls a multitude of processes, including cell shape and integrity, division, movement, and intracellular transport [33]. Actin is a highly conserved protein in eukaryotic cells byplaying important roles in muscle contraction, cell motility, cytoskeletal structure, and cell differentiation [31]. During early development, massive rearrangements of actin networks occur in the beginning and continue throughout the development in response to intracellular signals [34], [35]. Embryonic myosin heavy chain is implicated in the formation of different organs; two isoforms of myosin were expressed exclusively in the ova and one isoform in the larval stage. ISS play an essential role as a motor protein to generate a force for muscle contraction by interacting with actin filaments [36], [37]. Previous studies reported an abundance of cytoskeleton proteins in the larval stages of polycheates species; *P.vexillosa* [11] and *H.elegans* [13]. A similar trend of cytoskeleton proteins (TUB, ACT and ISS) abundance and its isoforms distribution in larval stages was observed in the present study. This implies that polychaete share common early developmental transitions, including the loss of larval structures and cellular differentiation [38]. The CP is primary microtubule-organizing centers and facilitates spindle assembly from spindle poles during mitosis. The specific expression of CPs in early larval stage indicates microtubule-nucleating activity [39]. These findings suggest the existence of possible links between the identified cytoskeleton proteins during egg to larval transition.

### Protein Phosphorylation Dynamics of Ova and Larval Proteins

Phosphorylation of egg proteins are immediate regulatory devices used at egg activation and development. The twelve phosphoproteins identified have a role in protein synthesis, cell division and metabolism. A gradual increase in tubulin and actin phosphorylation during ova to larval stages was observed suggesting a role for phosphorylation in remodeling of the cytoskeleton proteins. The detection and identification of HSP60 in abundance in all the three stages suggests the role of molecular chaperones during early development. HSP are involved in cell maintenance and protein biosynthesis. HSP genes are expressed in the early developmental stages where they stabilize proteins against environmental stresses [40]. Only a few previous studies have documented the presence of HSPs during early developmental stages [41], [42]. HSP60 acts as molecular chaperones for protein folding [43] and is involved in repairing protein damage that occurs as a consequence of environmental stress. The expression and abundance of this identified cell defense protein could overcome stress resistance. The EMBP is a multifunctional, fibronectin-binding cell surface protein that mediates attachment to host extracellular matrix, biofilm accumulation and escape from phagocytosis [44]. The phosphorylation and specific expression of EMBP isoforms in ova and larvae may be involved in cell attachment to the extracellular matrix. Since this study focused only on the most abundant proteins, all identified proteins are highly conserved across the animal kingdom due to their functional constraints in the cellular maintenance. However, abundant proteins or its isoforms may be important as their differential or specific expression may indicate important changes in biological functions.

Phosphorylated proteins/peptides from larval samples were identified by two complementary proteomic methods; (1) gel-based 2-DE phosphoprotein identification, (2) gel-free enrichment of phosphopeptides on IMAC followed by LC-MS/MS. Few phosphopeptides were identified in 2-DE because of the low abundance of phosphoproteins. However, an attempt was made to establish a gel-free proteomic protocol with the goal of improving phosphopeptides identification in complex proteome of polychaete larvae. Gel-free proteomics combined with IMAC enrichment proved to be superior in terms of phosphopeptide identification in contrast to a gel-based approach. This may afford a more effective method for the determination of in vivo phosphorylation sites in larval tissue extracts of marine polychaete. The cytoskeleton proteins phosphorylation may regulate cell and tissue assembly occur more frequently when larvae are ready to settle and metamorphose. These proteins forms core structure of the cilia and contribute to the ciliation in polychaetes [12], [45]. ATPase maintains steady level of ATP during early development by mitochondrial oxidative phosphorylation. The maintenance of oxidative phosphorylation by balancing ATP demand prevents mitochondrial damage by oxidative stress. Mitochondrial phosphorylation of ATPase in large number of eggs [46] may provide ATP during early development of larvae. Oxidation of mitochondrial pyruvate supplied ATP during early development [47]. The activity of the PD complex is regulated by reversible phosphorylation of serine residues. Isoenzymes of protein kinase differ in their abilities to phosphorylate the PD [48]. SIP is a signal-modifying enzyme that regulates PI3K signaling pathway and may affect development of fertilized eggs by inhibition of phosphorylated Akt at Ser473 [49]. Presently, it is not known how phosphorylation of CBR8, GK and MAD6 affects the activities and functions of these proteins during early development. Phosphorylation of proteins at Thr/Ser sites identified in this study is the first-reported observance in larvae of marine polychaetes. The specific regulatory mechanisms that account for phosphorylation at specific sites of proteins remain unknown and are to be investigated in the future.

In summary, proteomic analysis of the fertilized ova and larvae has yielded significant insight molecular changes in early developmental stages of *N*. *arenaceodentata.* Most of the proteins and phosphoproteins that occurred in high abundance were different isoforms of cytoskeleton proteins which suggests the probable role of microtubule dynamics and linkage during early development. The proteins identified serve as candidates for future investigation that may lead to a comprehensive analysis of phosphoproteomic changes that are required during the egg to larval transition. However, despite cross-species protein identification, we have only identified a fraction of the proteins and phosphoproteins because of high complexity of the sample. We believe that many of the proteins yet to be characterized are likely to be critical players.

## Supporting Information

Figure S1 **Replicate 2-DE gels of proteome and phosphoproteome of fertilized ova, and two larval stages of** ***Neanthes arenaceodentata*** **.** The gels indicates a technical reproducibility of 2-DE workflow and consistent pattern of protein and phosphoprotein spots between replicate gels.
(TIF)

Figure S2 **Representative tandem mass spectra of the phosphorylated peptide^ 625^YSGHSMSDPGTSYR^640^and ^285^MLFTPPEDTSPAGGKK^298^.** A: MS/MS spectrum of the peptide phosphorylated at Ser-291. B: MS/MS spectrum of the peptide phosphorylated at Thr- 628. The peptide fragment location within the protein is indicated by the NH2- and COOH-terminus of the residual phosphorylated peptide sequence.
(TIF)

Table S1 **Identification of abundant proteins during early development in** ***Neanthes arenaceoentata*** **by ESI-QTOF.** a) Accession numbers are from *Capitella* sp I* genome database and in-house transcriptome sequences of H.elegans** and *P.vexillosa*. *** Significance threshold level for positive identification was p<0.05. PM: number of peptides matched to protein sequence; SC: sequence coverage.
(DOCX)
