# Supplementary figures and images for: Gel-Based and Gel-Free Identification of Proteins and Phosphopeptides during Egg-to-Larva Transition in Polychaete Neanthes arenaceodentata
Source: PLoS One. 2012 Jun 15;7(6):e38814. doi: 10.1371/journal.pone.0038814 (PMC3376139; doi:10.1371/journal.pone.0038814)

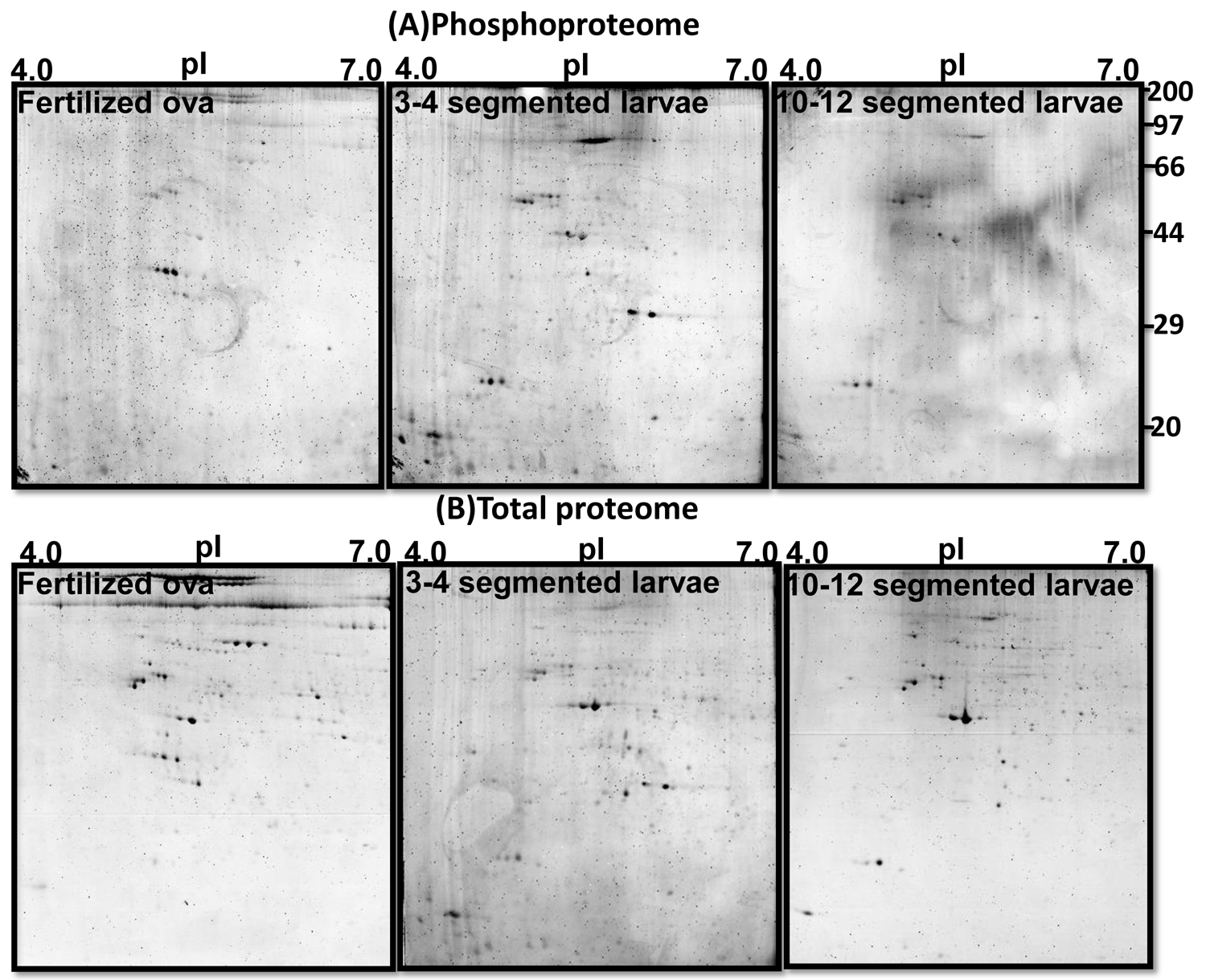

Supplement: Figure S1 — Replicate 2-DE gels of proteome and phosphoproteome of fertilized ova, and two larval stages of Neanthes arenaceodentata . The gels indicates a technical reproducibility of 2-DE workflow and consistent pattern of protein and phosphoprotein spots between replicate gels. (TIF) [file pone.0038814.s001.tif]

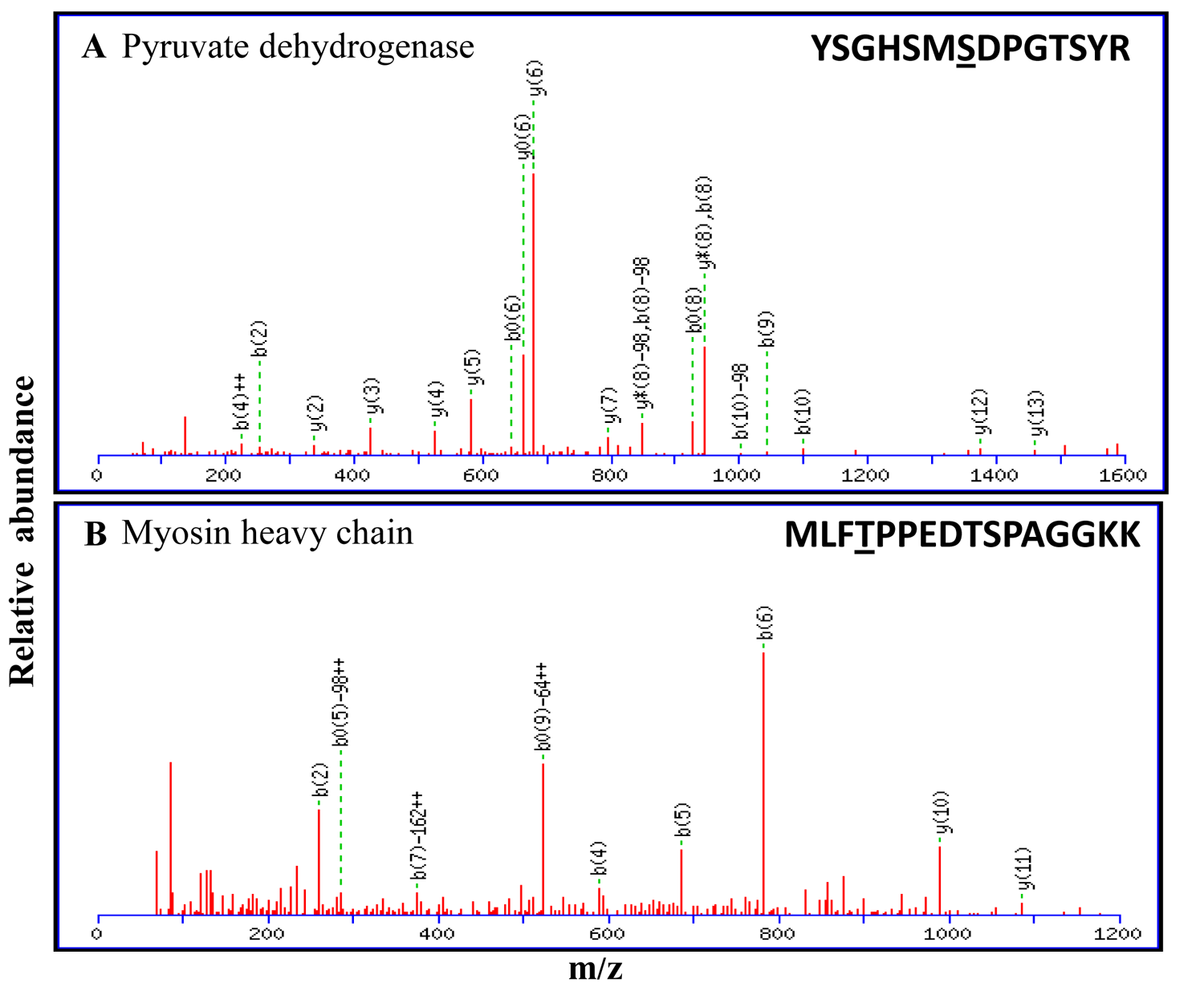

Supplement: Figure S2 — Representative tandem mass spectra of the phosphorylated peptide 625YSGHSMSDPGTSYR640and 285MLFTPPEDTSPAGGKK298. A: MS/MS spectrum of the peptide phosphorylated at Ser-291. B: MS/MS spectrum of the peptide phosphorylated at Thr- 628. The peptide fragment location within the protein is indicated by the NH2- and COOH-terminus of the residual phosphorylated peptide sequence. (TIF) [file pone.0038814.s002.tif]
