# Supplementary material for: Gel-Based and Gel-Free Identification of Proteins and Phosphopeptides during Egg-to-Larva Transition in Polychaete Neanthes arenaceodentata
Source: PLoS One. 2012 Jun 15;7(6):e38814. doi: 10.1371/journal.pone.0038814 (PMC3376139; doi:10.1371/journal.pone.0038814)
Supplement: Table S1 — Identification of abundant proteins during early development in Neanthes arenaceoentata by ESI-QTOF. a) Accession numbers are from Capitella sp I* genome database and in-house transcriptome sequences of H.elegans** and P.vexillosa. *** Significance threshold level for positive identification was p<0.05. PM: number of peptides matched to protein sequence; SC: sequence coverage. (DOCX) [file pone.0038814.s003.docx]

| **Spot**  **No ^a)^.** | **Acc No.** | **Protein name** | **MW(Da)** | **pI** | **PM/SC** |
| --- | --- | --- | --- | --- | --- |
| 4** | \| [GF03LTE04I7KPH_3](http://lfe0018/mascot/cgi/master_results.pl?file=../data/20111020/F001732.dat#Hit1) \|  \| \| --- \| --- \| | hypothetical protein | 15306 | 6.60 | 1/5 |
| 8** | Isotig01855_7 | non-muscle actin II | 42059 | 5.29 | 6/21 |
| 8* | Jgi\|Capca1\|158679 | actin, cytoplasmic 1 | 41983 | 5.30 | 7/26 |
| 10*** | [GF03LTE04I7KPH_3](http://lfe0018/mascot/cgi/master_results.pl?file=../data/20111020/F001732.dat#Hit1) | hypothetical protein | 20000 | 5.70 | 15/14 |
| 11*** | F5K2Q4C01CF4OT_4 | actin | 9360 | 5.61 | 2/21 |
| 14** | Isotig15569_1 | heat shock protein 60 | 16967 | 4.82 | 1/13 |
| 14* | Jgi\|Capca1\|179778 | heat shock protein 60 | 62475 | 5.19 | 2/5 |
| 15*** | GG7OGXE04JX7QT_8 | beta-tubulin | 4630 | 8.37 | 1/28 |
| 15** | Isotig14472_10 | beta-tubulin | 50348 | 4.75 | 4/10 |
| 15* | Jgi\|Capca1\|225661 | beta-tubulin | 48784 | 5.44 | 5/14 |
| 17*** | F5K2Q4C01CF4OT_4 | actin | 9360 | 5.61 | 2/21 |
| 17** | Isotig01855_7 | non-muscle actin II | 42059 | 5.29 | 5/13 |
| 17* | Jgi\|Capca1\|158679 | actin, cytoplasmic 1 | 41983 | 5.30 | 7/18 |
| 18*** | Isotig01330_11 | beta-actin | 13912 | 7.82 | 3/28 |
| 18** | Isotig01855_7 | non-muscle actin II | 42059 | 5.29 | 6/25 |
| 18* | Jgi\|Capca1\|160805 | actin | 40444 | 5.66 | 8/37 |
| 19*** | GG7OGXE04JX7QT_8 | tubulin | 4630 | 8.37 | 1/38 |
| 19** | Isotig14472_10 | tubulin | 50348 | 4.75 | 9/30 |
| 19* | Jgi\|Capca1\|225661 | tubulin | 48784 | 5.44 | 9/31 |
| 20*** | F5K2Q4C01CF4OT_4 | actin | 9360 | 5.61 | 1/12 |
| 20** | Isotig01855_7 | non-muscle actin II | 42059 | 5.29 | 1/2 |
| 20* | Jgi\|Capca1\|158679 | actin, cytoplasmic 1 | 41983 | 5.30 | 2/5 |
| 21*** | F5K2Q4C01CF4OT_4 | actin | 9360 | 5.61 | 2/21 |
| 21** | Isotig01855_7 | non-muscle actin II | 42059 | 5.29 | 5/16 |
| 21* | Jgi\|Capca1\|158679 | actin, cytoplasmic 1 | 41983 | 5.30 | 5/21 |
